# Survival and associated factors in 268 adults with Pompe disease prior to treatment with enzyme replacement therapy

**DOI:** 10.1186/1750-1172-6-34

**Published:** 2011-06-01

**Authors:** Deniz Güngör, Juna M de Vries, Wim CJ Hop, Arnold JJ Reuser, Pieter A van Doorn, Ans T van der Ploeg, Marloes LC Hagemans

**Affiliations:** 1 Center for Lysosomal and Metabolic Diseases, Erasmus MC University Medical Center, Dr. Molewaterplein 60, 3015 GJ Rotterdam, the Netherlands; 2 Department of Pediatrics, Erasmus MC University Medical Center, Dr. Molewaterplein 60, 3015 GJ Rotterdam, the Netherlands; 3 Department of Neurology, Erasmus MC University Medical Center, 's-Gravendijkwal 230, 3015 CE Rotterdam, the Netherlands; 4 Department of Biostatistics, Erasmus MC University Medical Center, Dr. Molewaterplein 50-60, 3015 GE Rotterdam, the Netherlands; 5 Department of Clinical Genetics, Erasmus MC University Medical Center, Dr. Molewaterplein 50-60 3015 GE, Rotterdam, the Netherlands

**Keywords:** Pompe disease, survival, acid maltase deficiency, lysosomal storage disease, glycogen storage disease type II, prognostic factors, natural course, patient reported outcome measures

## Abstract

**Background:**

Pompe disease is a rare lysosomal storage disorder characterized by muscle weakness and wasting. The majority of adult patients have slowly progressive disease, which gradually impairs mobility and respiratory function and may lead to wheelchair and ventilator dependency. It is as yet unknown to what extent the disease reduces the life span of these patients. Our objective was to determine the survival of adults with Pompe disease not receiving ERT and to identify prognostic factors associated with survival.

**Methods:**

Data of 268 patients were collected in a prospective international observational study conducted between 2002 and 2009. Survival analyses from time of diagnosis and from time of study entry were performed using Kaplan-Meier curves and Cox-proportional-hazards regression.

**Results:**

Median age at study entry was 48 years (range 19-79 years). Median survival after diagnosis was 27 years, while median age at diagnosis was 38 years. During follow-up, twenty-three patients died prior to ERT, with a median age at death of 55 (range 23-77 years). Use of wheelchair and/or respiratory support and patients' score on the Rotterdam Handicap Scale (RHS) were identified as prognostic factors for survival. Five-year survival for patients without a wheelchair or respiratory support was 95% compared to 74% in patients who were wheelchair-bound and used respiratory support. In a Dutch subgroup of 99 patients, we compared the observed number of deaths to the expected number of deaths in the age- and sex-matched general population. During a median follow-up of 2.3 years, the number of deaths among the Dutch Pompe patients was higher than the expected number of deaths in the general population.

**Conclusion:**

Our study shows for the first time that untreated adults with Pompe disease have a higher mortality than the general population and that their levels of disability and handicap/participation are the most important factors associated with mortality. These results may be of relevance when addressing the effect of ERT or other potential treatment options on survival.

## Background

Pompe disease, synonymously 'acid maltase deficiency' or 'glycogen storage disease type II', is a metabolic myopathy caused by deficiency of the enzyme acid alpha-glucosidase and resulting in intralysosomal accumulation of glycogen. This autosomal recessive disorder is mainly characterized by progressive loss of muscle strength and respiratory function due to destruction of muscle tissue [1,2]. Because of its low frequency of approximately 1 in 40,000 births and the broad ethnic spreading [3-5], Pompe disease is a true orphan disease with the associated problem of collecting data in sufficiently large groups. Clinical heterogeneity is an additional complicating factor [5,6]. Classic infantile Pompe disease, the most severe form, presents in the first months of life with generalized muscle weakness and cardiac hypertrophy. Without treatment these infants die before age one. Later-onset forms of Pompe disease comprise childhood, juvenile, and adult cases. The majority of these patients present with symptoms in adulthood with limb- girdle weakness and respiratory problems [5].

For a long time, supportive care such as respiratory support was the only way of managing Pompe disease, but in the course of 2006 enzyme replacement therapy (ERT) with recombinant human alpha-glucosidase became available. Clinical trials showed that ERT can ameliorate motor outcome, improve cardiomyopathy and prolong survival in classic infantile Pompe disease [7-11]. In children and adults treatment with ERT has been shown to stabilize respiratory function and to improve muscle function [12-16].

In contrast to classic infantile Pompe disease, in which survival is a key outcome measure to describe the natural course of the disease *and *to evaluate the effects of treatment, information on mortality in adults with Pompe disease has been lacking. The present study was performed to fill the gap of knowledge on the impact of Pompe disease on survival in untreated adult patients, using data collected prospectively in an international patient survey prior to the introduction of ERT. The objective was to determine natural course survival in adult patients with Pompe disease, to compare this to the general population and to assess differences in survival between subgroups of patients.

## Methods

### Data

Data were collected between May 2002 and December 2009 as part of an ongoing study on the natural course of Pompe disease ('Pompe Survey') e.g. [6,17] in which patients complete a number of self-report questionnaires each year, gathering information on medical history, current disease status, use of care and quality of life.

Patients were recruited through patient organizations affiliated with the International Pompe Association (IPA) in Australia, Canada, Germany, the Netherlands, the United Kingdom and the United States. Inclusion criteria for the Pompe Survey were a diagnosis of Pompe disease and an age above 2 years. The present analyses only include patients of 18 years and older with late-onset Pompe disease.

For the Dutch patients, more information was available as Erasmus MC was designated as the single referral center for treatment and longitudinal follow-up of Pompe patients in the Netherlands.

All research protocols were approved by the medical ethics committee of Erasmus MC and/or the Central Committee on Research Involving Human Subjects. Written informed consent was obtained from all patients.

### Explanation of variables

For the international participants in the Pompe Survey, the date of the last completed questionnaire before December 2009 was considered as the date of last follow-up. For the Dutch subgroup, the date of last follow-up was the last visit at our hospital in 2009 or the date of the last completed questionnaire, whichever came last.

When the date of death for the deceased was not exactly known it was estimated to be halfway between the date of the last completed questionnaire and the date at which the next questionnaire should have been completed.

The date of diagnosis was estimated as precisely as possible according to the information provided in the questionnaires.

To assess the level of participation (defined as a person's involvement in life situations; previously called 'Handicap') [18,19], the Rotterdam Handicap Scale (RHS) was used. The RHS consists of 9 questions on the topics of mobility, kitchen tasks, domestic tasks, leisure activities, travelling and work or study. The scores per item range from 1 ('unable to fulfil the task or activity') to 4 ('complete fulfilment of the task or activity'). If an item is not applicable to a patient, a score of 0 is given. The total score is calculated as the sum of the scores per item * 9/(9-number of non-applicable or missing items) [17,20]. The RHS score thus ranges from 9 to 36 and in the present analysis the number of items necessary to calculate a score was 5 out of 9 questions.

To assess disability level at study entry patients were divided into four groups: 1) no wheelchair or respiratory support, 2) only wheelchair, 3) only respiratory support and 4) both wheelchair and respiratory support. No division was made between partial and fulltime respiratory support, or whether it was invasive or non-invasive.

According to their nationality patients were divided into the following groups: Netherlands, United Kingdom, United States, Germany, Canada, Australia and other (Denmark, Austria, Switzerland, Spain, Italy, New Zealand, Greece, Taiwan and Luxembourg).

### Statistical analysis for survival from diagnosis and from study entry

Survival was calculated from the date of diagnosis or study entry until the date of last follow-up, start of ERT or death. The survival times of patients who were alive at study end or lost to follow-up were censored. The survival times of the patients were also considered censored at the initiation of ERT.

For survival from diagnosis and from study entry, the influence on survival was tested for the following variables: gender, age at diagnosis, and nationality. The variables age at entry, disability level and RHS score were only tested for survival from study entry.

For survival from diagnosis, the PROC PHREG method in SAS was used, because most of our patients were enrolled months or years after diagnosis. This means that the data are 'left-truncated', as opposed to usual time-to-event data where all patients are followed from diagnosis. Estimates of survival from diagnosis in case not all patients enter the cohort study at the time of diagnosis require special calculations as described by Kurtzke [21].

Univariate analysis for survival from study entry was estimated by using the Kaplan-Meier method. Factors influencing survival were identified with the log-rank test. Multivariate analysis was performed with the Cox proportional-hazards method.

### Mortality of Dutch patients compared to the general population

Death probabilities from study entry were compared between the Dutch Pompe patients and the general population using death probabilities derived from the Dutch Central Bureau of Statistics (CBS) [22]. For each case of our study population, the death probability per follow-up year of someone of the same age and gender from the general population was taken for comparison. Annual death probabilities per person were summed up and the sum of these cumulative death probabilities of the matched persons from the general population was used as the expected number of deaths. This was then compared to the observed number of deaths in our own cohort using the Poisson distribution.

All analyses were performed using SAS (version 9.2) or SPSS (version 15.0). Statistical significance was defined as a p-value ≤ 0.05 for all analyses.

## Results

### Patient characteristics

As of December 2009, 303 adult patients were enrolled in the Pompe Survey. Thirty-five of them were excluded from the analyses. These were patients who had provided too little information about their diagnosis (n = 8), patients with only baseline data available (n = 15), patients already receiving ERT at study entry (n = 2) and patients with important data missing such as date of birth (n = 10). Thus, the current analyses covering the years 2002 to 2009 comprise a total of 268 adults with Pompe disease from 15 different countries. Patient characteristics are summarized in Table 1.

**Table 1:** Patient Characteristics of 268 untreated adult patients with Pompe disease

Female, n (%)	141 (53)
Median age at study entry, years (range)	48 (19-79)
Median age at diagnosis, years (range)	38 (1-68)
Number of patients diagnosed in age categories of 15 years, n (%)	
<15 years	22 (8)
16-30 years	59 (22)
31-45 years	115 (43)
46-60 years	61 (23)
>61 years	11 (4)
Nationality, n (%)	
Netherlands	99 (37)
Germany	48 (18)
US	69 (26)
UK	20 (8)
Australia	13 (5)
Canada	9 (3)
Other	10 (4)
Median disease duration at entry, years (range)	9 (0-32)
Median follow up time, years (range)	3.5 (0.02-7)
Disability level at study entry, n (%)	
No wheelchair use or respiratory supportª	127 (47)
Wheelchair use	34 (13)
Use of respiratory support	39 (15)
Both wheelchair use and respiratory support	68 (25)
Median RHS score* at study entry (range) (n = 258)	27 (9-36)

Continuous variables are expressed as median (range). Categorical variables are expressed as n (%). ª'Respiratory support' includes partial and fulltime, invasive and non-invasive respiratory support *RHS assesses the level of participation/handicap; score varies between 9 (severe participation restrictions) and 36 (no participation restrictions).

At study entry patients' age varied between 19 and 79 years with a median age of 48 years. This did not differ significantly between countries. The median age at diagnosis was 38 (range 1-68) years. Median follow-up time from study entry was 3.5 years, with a maximum of 7 years. Seventy-eight percent of the patients were followed for 2 years or more and 62% of the patients for 3 years or more. Differences in disability level between countries were found, with the lowest rates of wheelchair and ventilator use in the Dutch patient group (32% and 26%, respectively). Almost all Dutch patients carried the most common *c.-32-13T >G (IVS1) *GAA mutation in combination with a fully deleterious mutation on the other allele. The *c.-32-13T>G (IVS1) *is a splice-site mutation leading to 10-20% residual activity of acid alpha-glucosidase and a broad clinical spectrum [23].

### Mortality

For 34 of the 268 patients, a death confirmation was received from the patient organization or the family. The median age at death was 56 years and did not differ significantly between countries. In 23 of the 34 cases information on cause of death was not available. For the Dutch patients (n = 9), causes of death were reported as respiratory insufficiency (n = 3), myocardial infarction (n = 2), aortic dissection (n = 1) and breast cancer (n = 1). For two of them, cause of death was not known. Characteristics of the deceased patients are listed in Table 2. Data from only 23 of the deceased patients (median age at death 55 years) could be used to estimate survival prior to ERT, since the other 11 received ERT prior to their death.

**Table 2:** Characteristics of deceased patients (34 patients died in total, 23 of them prior to ERT)

	34 (All deceased patients)	23 (died prior to ERT)
Female, n (%)	16 (47)	12 (52)
Median age at study entry, years (range)	54 (20-75)	51 (20-75)
Median age at death, years (range)	56 (23-78)	55 (23-77)
Median age at diagnosis, years (range)	42 (13-66)	42 (13-59)
Median disease duration, years (range)	14 (2-27)	16 (2-27)
Age at diagnosis in categories of 15 years, n (%)		
0-15 years	3 (9)	3 (13)
16-30 years	6 (18)	3 (13)
31-45 years	13 (38)	10 (44)
46-60 years	10 (29)	7 (30)
>61 years	2 (6)	0
Nationality, n (%)		
Netherlands	9 (27)	5 (22)
Germany	4 (12)	4 (17)
US	13 (38)	8 (35)
UK	4 (12)	3 (13)
Australia	1 (3)	0
Canada	2 (6)	2 (9)
Other	1 (3)	1 (4)
Disability level at study entry, n (%)		
No wheelchair use or respiratory support ª	4 (12)	4 (17)
Wheelchair use	6 (18)	4 (17)
Use of respiratory support	7 (21)	4 (17)
Both wheelchair use and respiratory support	17 (50)	11 (48)
Median RHS score* at study entry (range)	23 (9-36) (n = 33)	22 (9-36)

Continuous variables are expressed as median (range). Categorical variables are expressed as n (%). ª 'Respiratory support' includes partial and fulltime, invasive and non-invasive respiratory support. * RHS assesses the level of participation/handicap; score varies between 9 (severe participation restrictions) and 36 (no participation restrictions.

### Survival from diagnosis

**Figure 1:**
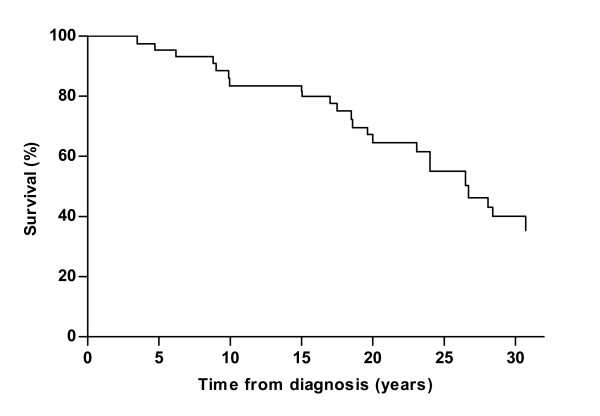
**Survival estimates of 268 ***untreated ***adults with Pompe disease from diagnosis until end of study, start of ERT or death**. Twenty-three patients died during follow-up.

The estimated median survival after diagnosis -without enzyme replacement therapy- was 27 years. The estimated 5-year survival after diagnosis was 95%. At 10, 20 and 30 years this was 83, 65 and 40%, respectively (Figure 1).

Univariate and multivariate analysis showed that none of the tested factors were related to survival after diagnosis.

Because the time between the start of ERT and death was mostly less than one year and these patients were already severely affected when they started ERT, we performed a second analysis including all patients who died within 18 months after start of ERT (n = 9). To be consistent with the deceased patients, the follow-up time for all other patients on ERT was also extended by 18 months. In this analysis with 32 events, only age at diagnosis, accounting for gender and nationality, was related to survival (Hazard Ratio 1.55 per 10 years of age P < 0.01).

### Survival from study entry

**Figure 2:**
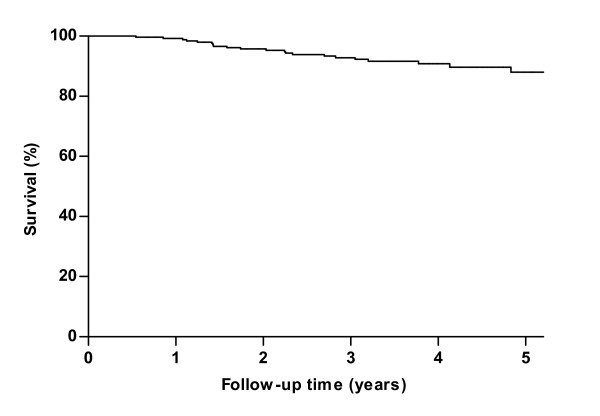
**Kaplan Meier survival estimates of 268 ***untreated ***adults with Pompe disease from study entry until end of study, start of ERT or death**. Twenty-three patients died during follow-up.

The Kaplan-Meier survival curve from study entry is shown in Figure 2. After 5 years of follow-up, 88% of the patients not yet receiving ERT were still alive.

**Figure 3:**
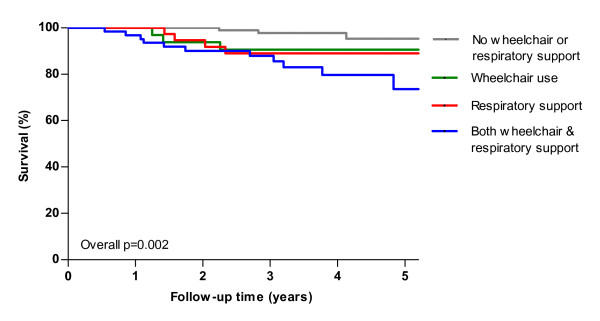
**Kaplan Meier survival estimates of 268 ***untreated ***adults with Pompe disease from study entry until end of study, start of ERT or death by disability level**. Twenty-three patients died during follow-up. 'Respiratory support' includes partial and fulltime, invasive and non-invasive respiratory support. P-value denotes result from log-rank test for trend.

**Figure 4:**
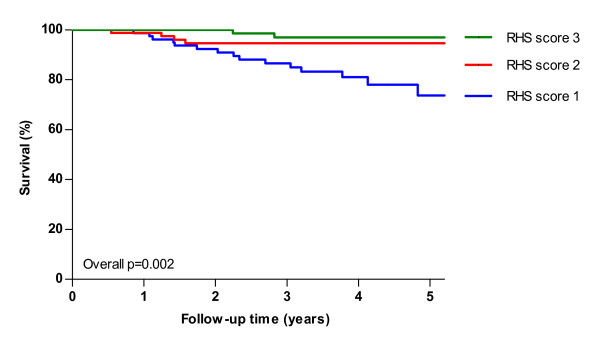
**Kaplan Meier survival estimates of 268 ***untreated ***adults with Pompe disease from study entry until end of study, start of ERT or death by RHS score.** (RHS score was divided into tertiles for comparison. RHS1 = score <23, RHS2 = score 23-30, RHS3 = score >30). Twenty-three patients died during follow-up. P-value denotes result from log-rank test for trend.

Univariate analysis revealed a statistically significant difference in survival between groups based on disability level (overall p = 0.002 log-rank, Figure 3), RHS score (overall p = 0.002 log-rank, Figure 4) and age at study entry (overall p = 0.03 log-rank). After five years 95% of patients without a wheelchair or respiratory support survived, while this was only 74% for patients with both wheelchair and respiratory support at study entry. Table 3 shows the 5-year survival with respect to potential prognostic factors. Multivariate analysis of the factors age at study entry, gender, nationality and disability level showed a significant effect of disability level (p = 0.01), i.e. less disability at study entry was associated with better survival. Analyzing RHS score instead of disability level showed that a higher RHS score at study entry was also associated with better survival (p < 0.001). In the analysis including 32 deceased patients, the factors age at study entry (p = 0.01) and disability level (p = 0.002) were significantly related to survival. When RHS score was analyzed instead of disability level both age (p = 0.01) and RHS score (p < 0.001) were significantly associated with survival.

**Table 3:** Summaries of 5-year survival from study entry according to potential prognostic factors (23 deceased patients)

Prognostic factors	n	5-year survival percentages	P-value*
Gender			0.7
Women	141	86	
Men	127	91	
Age at diagnosis			0.4
0-15 years	22	81	
16-30 years	59	92	
31-45 years	115	89	
46-60 years	61	82	
>61 years	11	100	
Age at entry			0.03
18-30 years	32	91	
31-45 years	85	94	
46-60 years	104	89	
>61 years	47	77	
Nationality			0.7
Netherlands	99	90	
Germany	48	93	
US	69	85	
UK	20	67	
Other°	32	93	
Disability level at study entry			0.002
No wheelchair use or respiratory support ª	127	95	
Wheelchair use	34	91	
Use of respiratory support	39	89	
Both wheelchair use and respiratory support	68	74	
RHS score at study entry*			0.002
1	85	74	
2	85	95	
3	88	97	

* Log rank test for the Kaplan-Meier curves, overall p-values of univariate analyses. ° Due to small groups Canada and Australia were classified into the category "other". ª 'Respiratory support 'includes partial and fulltime, invasive and non-invasive respiratory support. *RHS score was divided into tertiles for comparison. **1) **< 23, **2) **23-30**, 3**)>30.

### Mortality of Dutch Pompe patients compared to the general Dutch population

For this part of the analyses 99 Dutch patients, with median age at entry 50 (range 24-79) years, were included. The Dutch subgroup included 5 deceased patients before start and 4 after start of ERT. The median age at death was 55 (range 41-78) years. Two of the patients who died after start of ERT had died during the first year of treatment; one other had died 18 months after start of ERT and one had started and stopped ERT in the year before death. These were all severely affected patients using a wheelchair and/or respiratory support before start of ERT. To compare the probability of death in the Dutch Pompe patient group with that in the general Dutch population two analyses were performed: one taking into account only the 5 deaths before start of ERT (median follow-up time 2.3 years) and one taking into account 9 deaths, while in the latter analysis extending the follow-up time after start of ERT with 1 year for every patient on ERT (median follow-up time 3.3 years). Table 4 shows the results.

**Table 4:** Mortality of 99 Dutch Pompe patients compared to general Dutch population

Analysis	Median follow-up time (range)	Observed no. deaths (O)	Expected no. deaths (E) *	Ratio (O/E)	P -value
1	2.3 (<1 month-7 years)	5	2.3	2.2	0.09
2	3.3 (<2 months-7 years)	9	2.8	3.2	0.002

**Analysis 1: **5 deceased patients before start of ERT **Analysis 2: **including 4 patients who died after start of ERT with follow-up of every patient on ERT extended with 1 year after start of ERT. * According to death probabilities derived from Dutch Central Bureau of Statistics.

## Discussion

Using data from the Pompe Survey, a long-term, disease-specific database using patient-reported outcome measures, we were able to perform the first study on survival and prognostic factors in adults with Pompe disease.

Over a prospective follow-up period of 7 years 34 of 268 patients died, 23 of them prior to ERT. Some of these patients died relatively young (23 years) and some reached very high ages despite Pompe disease (78 years). By using cumulative death probabilities of persons from the general population matched by age and gender, our study shows for the first time that mortality in adults with Pompe disease is higher than in the general population.

We also found that in our group of patients, diagnosed at a median age of 38 years, 17% died ten years after diagnosis. The median (50%) survival was estimated at 27 years after diagnosis. In an earlier study on the relation between disease severity and disease duration based on Pompe Survey data, we showed that 10-15 years after diagnosis 50% of the patients were wheelchair-bound or ventilator dependent [24]. Thus, although Pompe disease in adults generally manifests as a slowly progressive disorder, it seriously affects the lives of patients.

Several factors in our study had a significant effect on survival. For patients without a wheelchair or respiratory support the 5-year survival from entry was 95%, while for patients with a wheelchair and respiratory support this was 74%. In practice this means that patients with a wheelchair and/or respiratory support have a shorter life expectancy at any age compared to patients without wheelchair and respiratory support. RHS score at study entry, indicative of the level of handicap or participation, also was significantly associated with survival. Whether the RHS score is also useful as a prognostic tool in clinical practice is a topic for further investigation.

The strength of our study is its prospective design, the regular follow-up, the representation across countries, and the large sample size, especially for a rare disorder such as Pompe disease. In orphan diseases, it is quite unique to be able to gather information on a large group of patients over so many years, especially prior to therapeutic intervention. Our prospective data collection was achieved by relying on patient reported outcome measures through a close collaboration with patient organizations. This approach enabled data collection without the support of a large physician's network that is -in orphan diseases-usually activated only after the introduction of a therapy. Our approach may stand model for data collection in other rare diseases. Since almost all newly diagnosed Pompe patients currently start with enzyme replacement therapy, this study might have been the very last chance to collect data on the natural course of Pompe disease.

Nevertheless, some methodological issues need further attention. Firstly, our patients were followed from 2002 onwards, which means that the majority entered the study at some cross-sectional point of their illness. The ideal method would have been to follow all patients from the time of disease onset or diagnosis until death. However, if we had applied those restrictions our study population would have been too small and the follow-up period would have been too short. Therefore, the next best thing was to also include the patients diagnosed before entering the study. This led to so-called 'left-truncated' data, with a grey area between diagnosis and study entry in which other patients may have died without entering our study, and could have caused an overestimation of the median survival. Additionally, because all data in the Pompe Survey are provided voluntarily, some deaths among enrolled patients who eventually became censored due to loss-to-follow-up (n = 37) may not have been reported.

Second, differences in wheelchair and ventilator use were observed between countries, with the Dutch patients tending to be less severely affected on average. This may be explained by the fact that the Dutch group includes almost all patients known in the Netherlands, while the inclusion through patient organizations in the other countries may have led to a larger proportion of more severely affected patients. This may have affected the estimation of median survival time, but does not influence our main conclusions that mortality in adults with Pompe disease is higher compared to the general population and is associated with disease severity.

Furthermore, our patients were followed for up to 7 years, but median follow-up time was 3.5 years. Although a longer follow-up of untreated patients would offer more insight in their survival, such a study will be difficult to do as most patients currently receive ERT.

Because our aim was to investigate the natural course survival, we censored patients at the initiation of ERT. This means that 11 patients who died *after *start of ERT were not included as deceased patients in our initial analysis. Most (n = 9) of these patients died within 1.5 year *after *start of ERT, or stopped ERT after a few infusions. As this treatment period is relatively short, we also performed analyses including these patients. Excluding these patients could have led to an underestimation of the number of deaths as these patients were already severely affected at the point they started ERT. All of them were wheelchair-bound and/or used respiratory support and most probably would also have died without ERT. For the same reason, in our comparison of death probabilities between the Dutch subgroup and the general population we also included the 4 patients who died shortly after start of ERT.

Unfortunately, information on cause of death was lacking for the majority of the deceased patients. However in our study, mortality was compared with the data obtained from the Dutch Central Bureau of Statistics, which reports deaths irrespective of the cause. This comparison showed that the difference in mortality between the two groups was statistically significant. This in itself is important information, which can be used to evaluate the severity of disease and may serve as a reference when comparing the mortality of patients under treatment. With regard to the reported causes of death, it seems likely that death due to respiratory insufficiency is related to Pompe disease [25,26]. Other causes, such as aortic dissection can also (in)directly be related to Pompe disease, as it may be a consequence of glycogen accumulation in vascular smooth muscle [27].

Whether timely start of ERT can increase survival of adults with Pompe disease is currently unknown. In a recently published randomized controlled trial of alglucosidase alfa in late-onset Pompe disease, significant differences in walking distance and pulmonary function between the alglucosidase alfa and placebo groups were found [16]. Considering these results, and given the fact that most patients die of respiratory failure, it might be expected that ERT will also positively influence life expectancy. The present study, in which we show that Pompe disease has a serious negative impact on the life span of untreated adult patients, allows for future evaluation of the effect of ERT with respect to this important parameter.

## Conclusion

Our study shows for the first time that mortality of untreated adults with Pompe disease is high compared to the general population. Both the need of a wheelchair and ventilator and a low RHS score are associated with higher mortality. Our results can serve as reference for future studies addressing survival of patients treated with ERT or alternative interventions. This information will also be valuable for families, genetic counsellors, and other health-care professionals when Pompe disease is diagnosed. Future studies should focus on identifying other factors -environmental or genetic- that may determine survival or disease progression in adults with Pompe disease, with or without treatment.

## Abbrevations

**CBS**: Dutch Central Bureau of Statistics; **ERT**: enzyme replacement therapy; **GAA**: the gene coding for acid alpha-glucosidase; **RHS**: Rotterdam Handicap Scale.

## Competing interests

The research on Pompe disease at Erasmus MC is financially supported by ZonMw- the Netherlands Organisation for Health Research and Development [project no. 152001005], the Dutch TI Pharma initiative "Sustainable Orphan Drug Development through Registries and Monitoring (T6-208), "EUCLYD-a European Consortium for Lysosomal Storage Diseases" (health F2/2008 grant agreement 201678), the Prinses Beatrix Fonds [project no. OP07-08], and Genzyme. As of August 2004, ATvdP and AJJR provide consulting services for Genzyme Corp, Cambridge, MA, USA, under an agreement between Genzyme Corp and Erasmus MC, Rotterdam, the Netherlands.

## Authors' contributions

DG was involved in study design and data collection, performed the statistical analyses and prepared the manuscript. JMdV was involved in data collection and critical revision of the manuscript for important intellectual content. WCJH supervised the statistical analysis, made substantial contribution to interpretation of the results, and critically revised the manuscript for important intellectual content. AJJR, PAvD and ATvdP were involved in study design and critical revision of the manuscript for important intellectual content. MLCH was involved in study design, data collection, statistical analyses, supervision throughout the study and critical revision of the manuscript for important intellectual content. All authors read and approved the manuscript.
